# Beyond the Lung: A Rare Presentation of Tuberculosis in the Tarsometatarsal Joint Treated With Chemotherapy, Surgical Debridement, and Fusion

**DOI:** 10.7759/cureus.76011

**Published:** 2024-12-19

**Authors:** Arun Sam, Saravana Kumar, Raja Shankar

**Affiliations:** 1 Orthopaedics and Traumatology, District Headquarters Hospital, Cuddalore, IND

**Keywords:** debridement and fusion, skeletal tuberculosis, surgery for foot tuberculosis, tuberculosis calcaneocuboid joint, tuberculosis foot

## Abstract

Foot tuberculosis is rarely reported in the literature, with most tuberculosis of the foot being an uncommon manifestation of skeletal tuberculosis. Early diagnosis and timely medical and surgical intervention can significantly reduce morbidity. A 23-year-old male presented with persistent swelling and pain in his right foot for six months, accompanied by a discharging sinus over the affected area in the last week, making weight-bearing increasingly difficult. Examination revealed cystic swelling near the cuboid bone, along with constitutional symptoms. Imaging studies, including X-ray and CT, showed osteopenia, bony erosions, and subluxation of the calcaneocuboid joint. MRI identified an abscess on the lateral aspect of the foot. Although aspiration and swab cultures were negative, the patient was empirically started on antitubercular therapy (ATT). Surgical debridement and fusion of the calcaneocuboid joint were performed using K-wires and tricortical bone grafts. A biopsy confirmed the diagnosis of tuberculosis. After completing nine months of ATT, the patient showed significant improvement, achieving a healthy scar and a functional plantigrade foot.

Biopsy and synovectomy, combined with chemotherapy, are essential treatments for skeletal tuberculosis. The interconnected nature of midfoot joints allows untreated osseous disease to spread, potentially causing joint instability, restricted movement, and poor functional outcomes. Early joint stabilization in the correct alignment is vital to prevent deformities. Similar to spinal tuberculosis management, radical debridement and bone grafting are effective in midfoot tuberculosis. Debridement reduces the infectious load, while bone grafting provides a stable framework to minimize recurrence and promote joint fusion. This approach not only alleviates pain but also enhances the patient’s functional recovery.

## Introduction

Tuberculosis (TB) predominantly targets the pulmonary system, but extrapulmonary forms are observed in about 15-20% of cases, with osseous TB affecting 10% of these patients [1]. Among those with bone involvement, nearly half experience spinal TB, most frequently in the lower thoracic region. Major weight-bearing joints like the hips and knees are less commonly affected, while TB of the hands and feet is rare. Around 90% of limb tubercular infections can typically be managed conservatively with medication, relative rest, and monitored rehabilitation. However, when diagnosis is delayed, rapid disease progression can lead to destructive changes that spread to adjacent joints, particularly in the foot. The primary diagnostic challenges involve the non-specific nature of imaging. While plain radiographs are typically the initial imaging method, MRI and CT scans offer more detailed views and can detect the disease earlier [1]. Microbiological tests have limited sensitivity and specificity due to the paucibacillary nature of TB lesions. Bone biopsy plays a crucial role in diagnosis by enabling histological evaluation for granulomas and facilitating sensitivity and resistance testing for antitubercular therapy (ATT) [1]. Surgical intervention may be necessary in cases with joint destruction, juxta-articular lesions, or insufficient local response to chemotherapy.

## Case presentation

**Figure 1:**
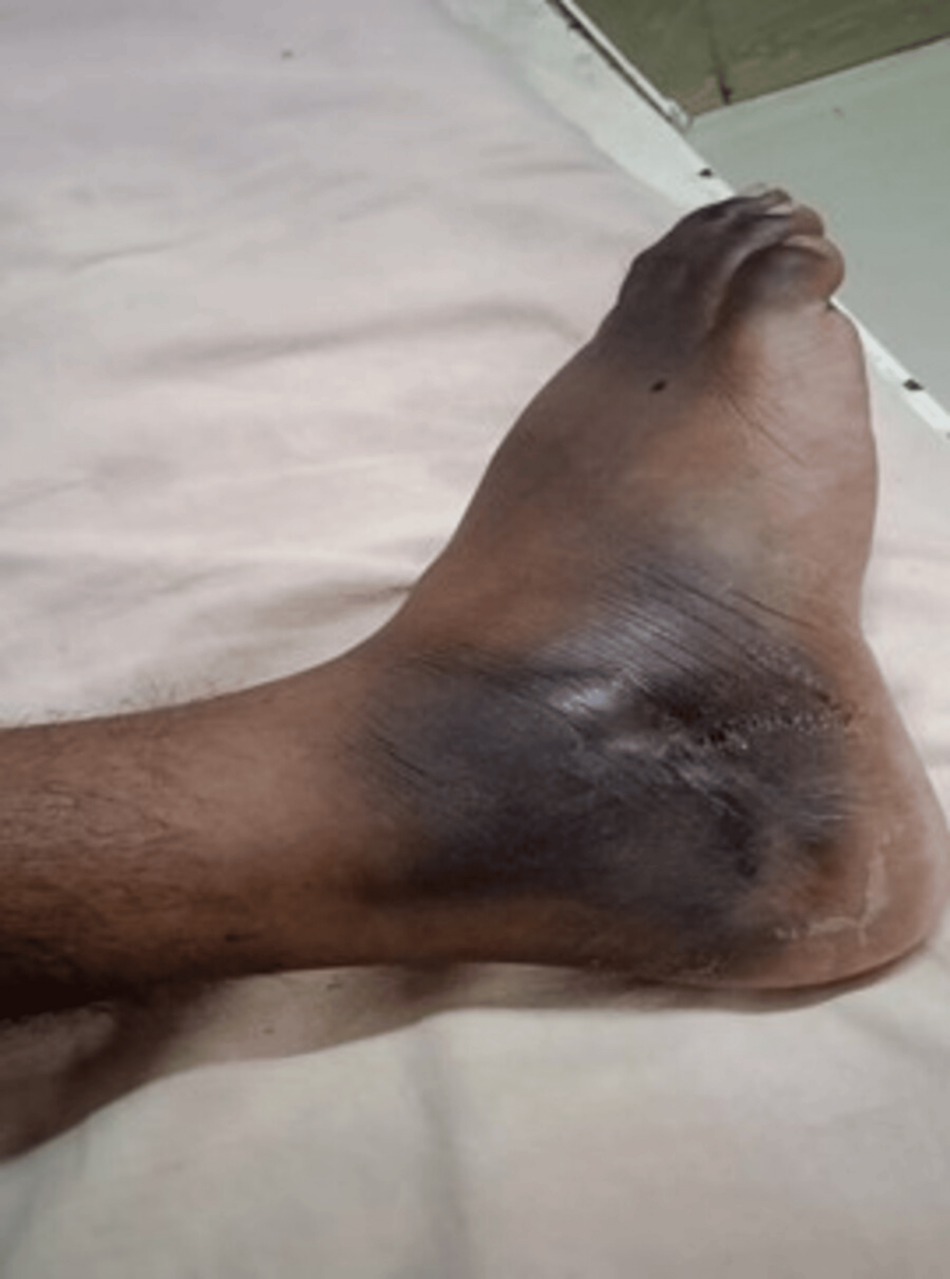
The image shows swelling over the anterolateral aspect of the right foot with skin hyperpigmentation noted along with a serous discharging sinus.

A 23-year-old male patient presented with swelling and pain in his right foot, persisting for the past six months. Over the past week, he developed a discharging sinus in the swollen area of his foot (Figure 1), making weight-bearing increasingly difficult. Examination revealed a 5 x 3 cm firm, cystic swelling over the cuboid bone. Approximately eight months prior, the patient sustained a fall, resulting in pain and diffuse swelling in the same area. This was initially treated as a ligament injury and eventually resolved. However, two months later, the current swelling reappeared and gradually increased in size. The patient reported a mild evening fever and reduced appetite. He also had a history of contact with a roommate who had pulmonary TB, six months prior.

Laboratory investigations revealed a mildly elevated C-reactive protein (CRP) level at 12.8 mg/L and an erythrocyte sedimentation rate (ESR) of 23 mm at 30 minutes and 49 mm at 60 minutes. The tuberculin skin test was positive, with a Mantoux reaction measuring 35 x 28 mm. Sputum samples for acid-fast bacilli (AFB) were negative. A chest radiograph showed a blunted costophrenic (CP) angle and haziness in the left lung.

**Figure 2:**
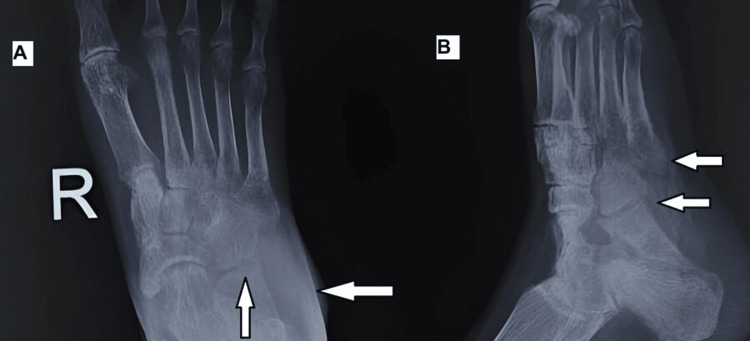
X-ray of the right foot in anteroposterior and oblique views. Image A (arrows) shows marginal erosion and soft tissue swelling. Image B (arrows) shows periarticular osteoporosis and joint space narrowing with subluxation of the cuboid.

**Figure 3:**
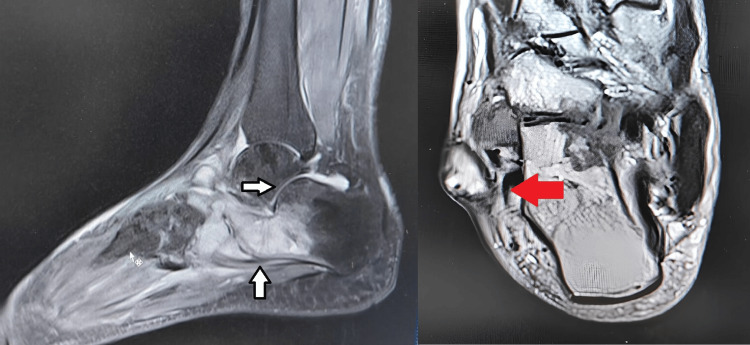
MRI of the right foot and ankle (sagittal cut and axial cut). Bone marrow signal changes were noted over the anterior calcaneum and cuboid, with fluid collection in the ankle joint. The red arrow shows a loculated abscess with granulation pointed out over the lateral aspect measuring 2 x 3 cm.

Local radiographs (Figure 2) revealed osteopenia in the cuboid and the bases of the 4th and 5th metatarsals, along with narrowing of the cuboid-metatarsal joint space. A CT scan of the right foot and ankle indicated a soft tissue collection in the lateral foot, subluxation of the calcaneocuboid joint, and sclerosis. MRI of the right foot and ankle (Figure 3) showed a loculated abscess with granulation tissue over the lateral foot and signal changes in the bone marrow of the anterior calcaneum and cuboid.

Aspiration and swab samples from the local site swelling were collected and sent for bacterial and fungal cultures, both of which returned negative. The sample was also tested with a cartridge-based nucleic acid amplification test (CBNAAT), yielding a negative result. Despite this, the patient was started on TB chemotherapy with a regimen of four fixed-dose combination (FDC) drugs per day, under suspicion of TB of the foot.

Due to persistent disability, particularly pain, even after three weeks of chemotherapy, the patient was admitted for synovectomy, curettage, and possible fusion of the tarsal and metatarsal joints, with bone grafting if necessary.

**Figure 4:**
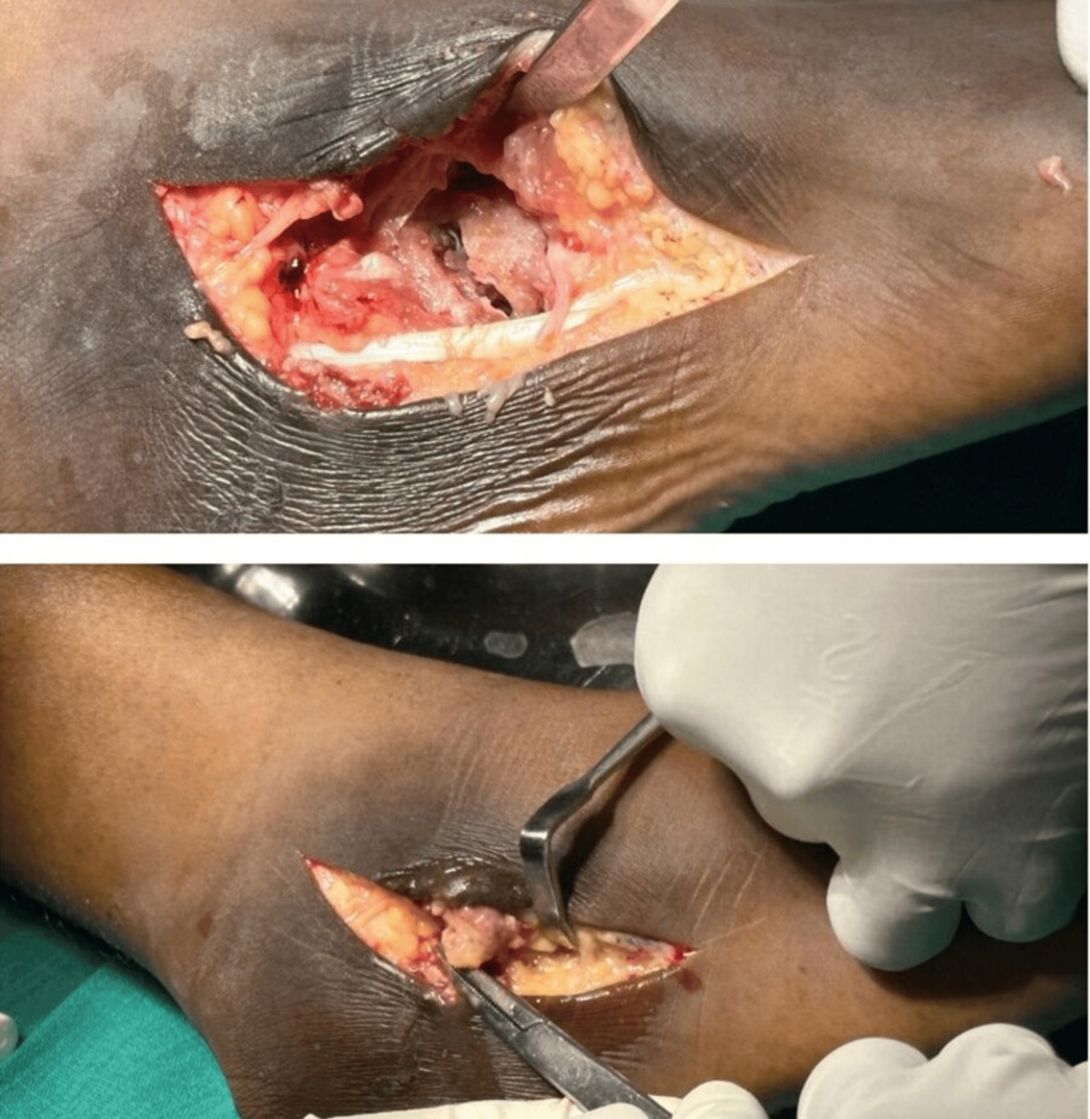
Intraoperative images. Marginal erosion was noted over the calcaneocuboid joint. Necrotic tissue over the anterolateral aspect was debrided.

An L-shaped incision was made along the lateral ankle and foot, extending from the lateral malleolus, encompassing the sinus and swelling, and following the calcaneocuboid joint, cuboid, and ending over the base of the 5th metatarsal. The sinus tract and synovial soft tissue swelling were excised (Figure 4) and sent for biopsy, microscopy, culture, and CBNAAT testing.

**Figure 5:**
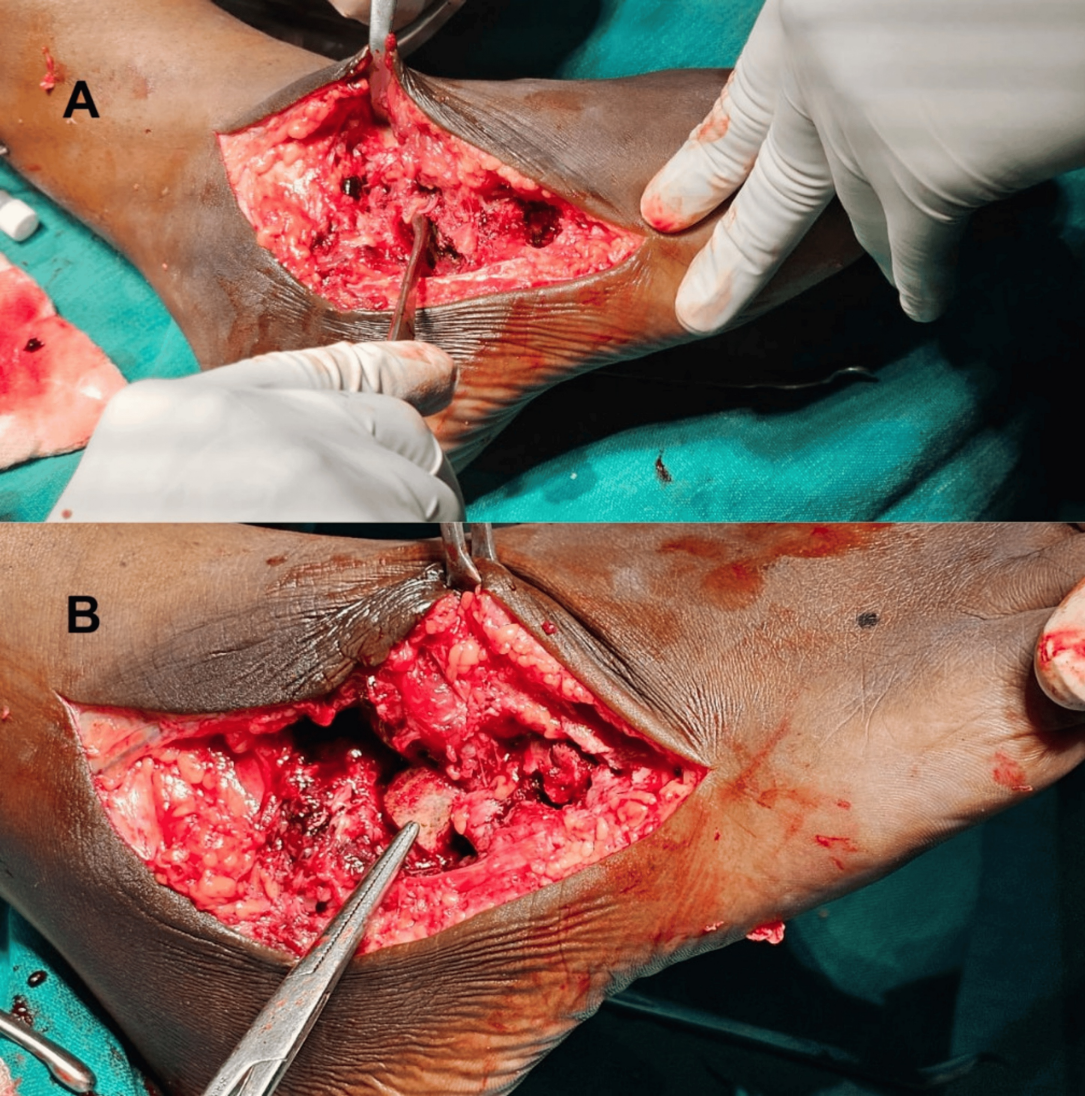
Intraoperative images. Image A depicts debridement of the affected area. Image B shows the placement of a tricortical bone graft in the unstable calcaneocuboid joint.

The articular cartilage of the cuboid bone was found to be separated and loose and was thoroughly curetted. The underlying bone appeared porotic and was also curetted (Figure 5A). Both the calcaneocuboid and cuboid-4th/5th metatarsal joint cartilages were affected and were carefully debrided. Drill holes were created in the anterior calcaneum and talar neck. Due to the significant void left in the calcaneocuboid and cuboid-metatarsal joints, these joints became further destabilized. To restore stability and maintain the length of the lateral column of the foot, the joints were fused using tricortical iliac bone autografts (Figure 5B) and secured with smooth K-wires. A plaster of Paris (POP) cast was applied, and the patient was advised to remain non-weight-bearing for three months, with continuation of TB chemotherapy.

Histopathological examination revealed necrotic tissue, granulation tissue, and vague granulomas, along with mixed cell infiltration consisting of lymphocytes, histiocytes, and multinucleated giant cells. These findings were suggestive of a necrotizing granulomatous inflammatory pathology. Culture results showed no growth, and Ziehl-Neelsen staining did not identify any acid-fast bacilli. Gram staining revealed moderate pus cells, but no organisms were observed. However, tissue CBNAAT detected the presence of *Mycobacterium tuberculosis*.

**Figure 6:**
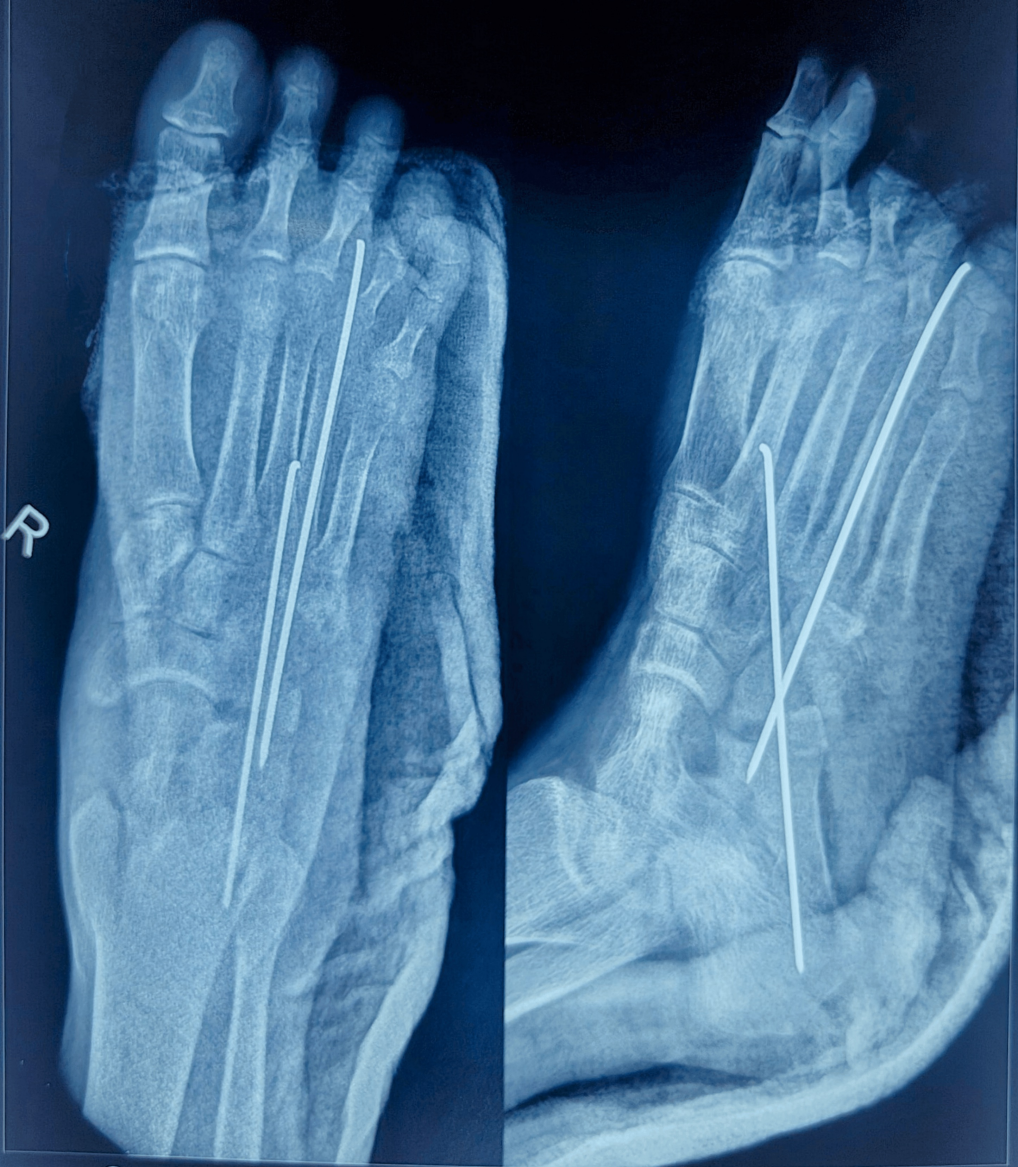
First month postoperative X-ray of the right foot in anteroposterior and oblique views, with K-wires in position protected by plaster of Paris (POP) below the knee slab.

**Figure 7:**
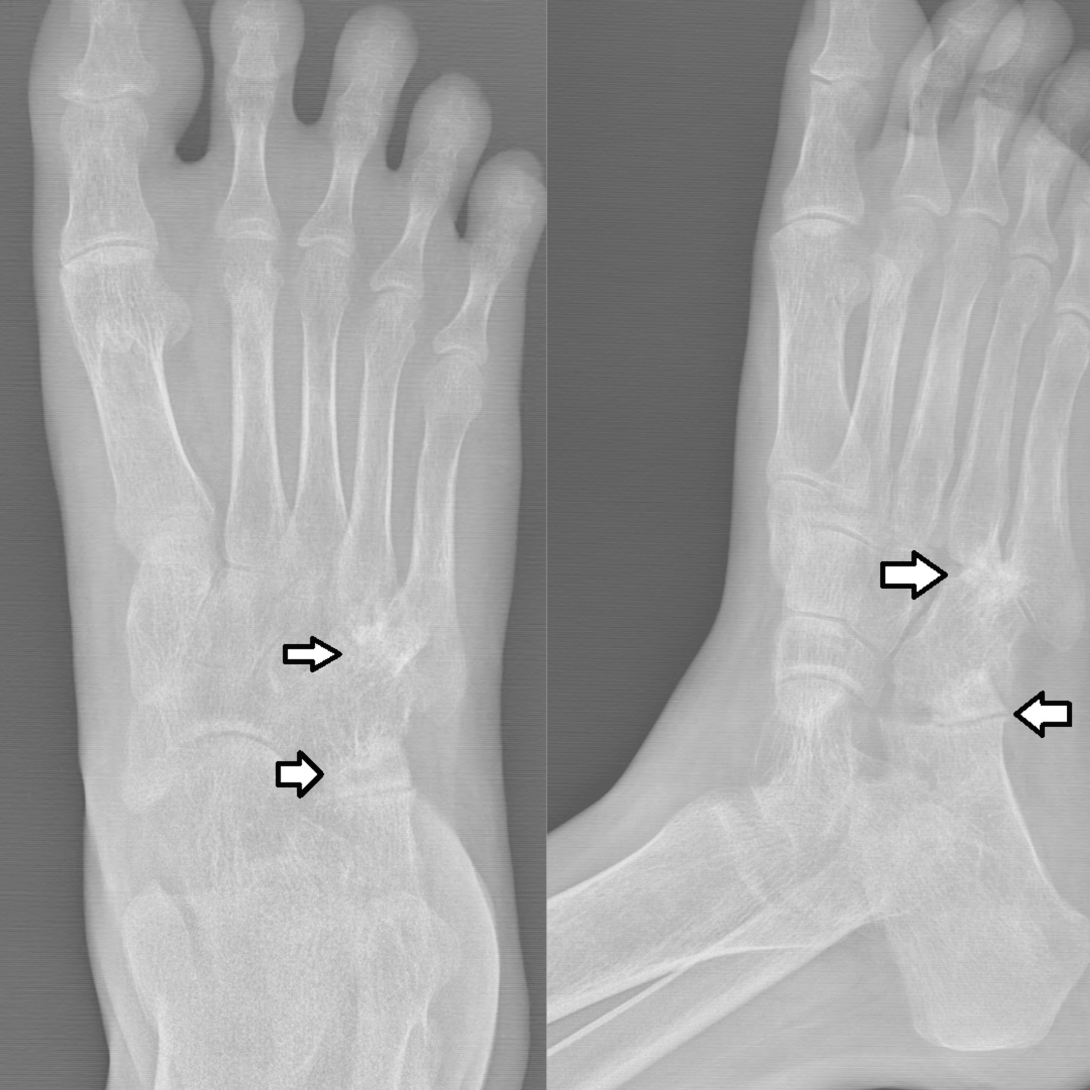
Conventional X-ray of the right foot in anteroposterior and oblique views taken six months post procedure, with arrows indicating bone graft consolidation.

**Figure 8:**
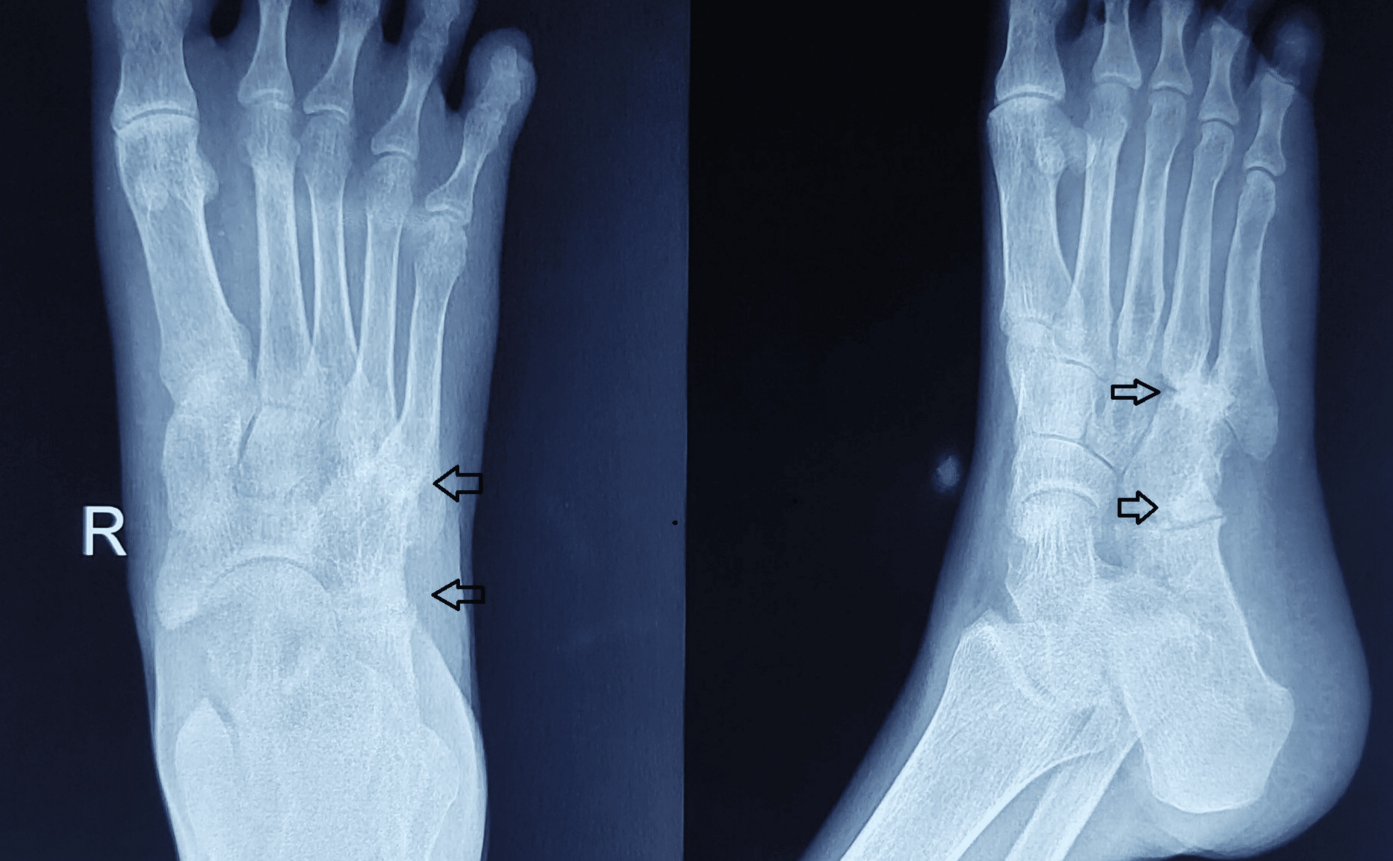
Postoperative follow-up X-rays at the 9th month show bone graft consolidation at the calcaneocuboid and tarsometatarsal joints.

**Figure 9:**
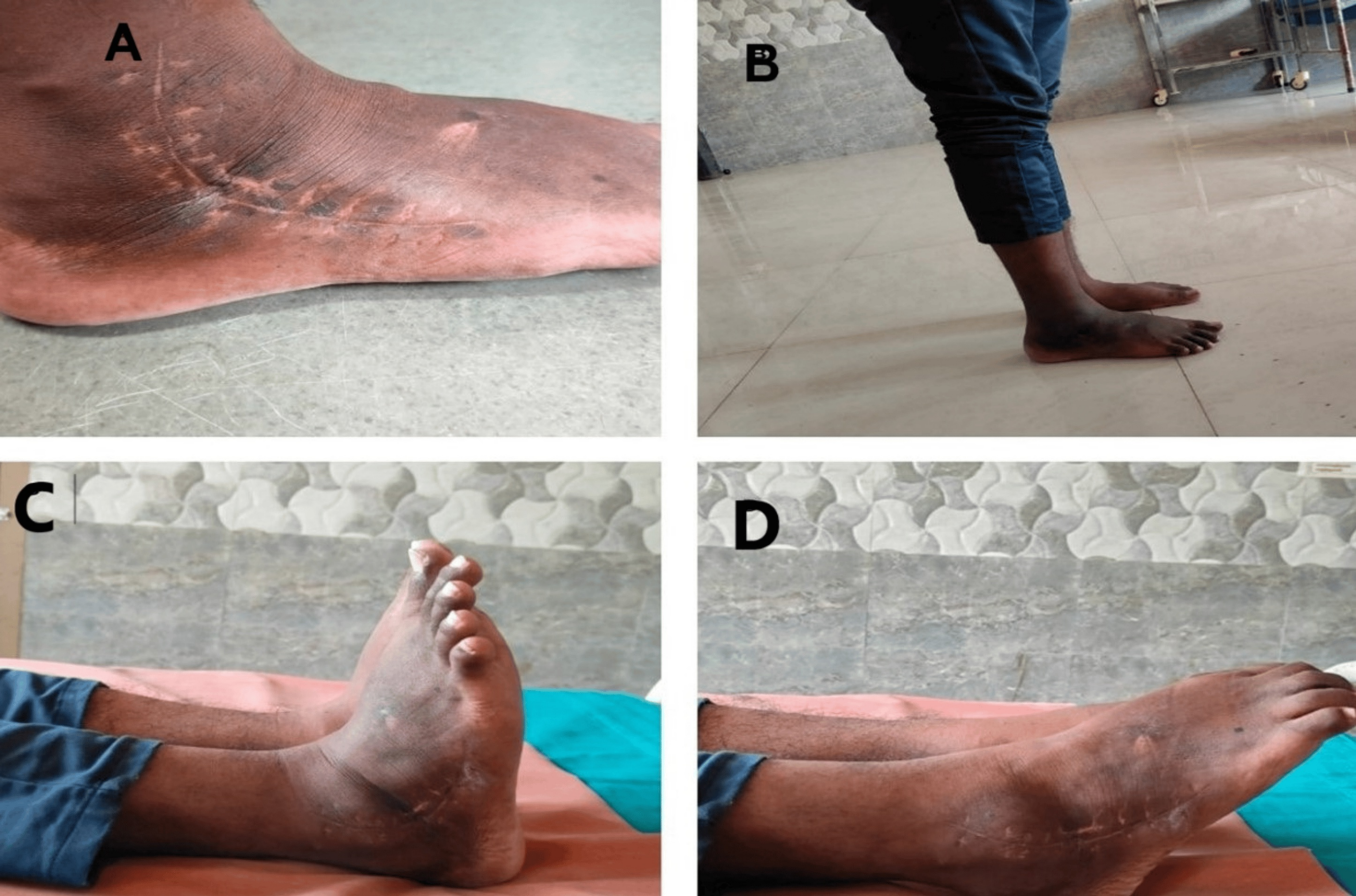
Clinical images. (A) Healed scar of the anterolateral approach. (B) Patient's ability to bear weight without support. (C) Good dorsiflexion. (D) Good plantar flexion.

The patient was regularly monitored for clinical progress, including dressing changes, suture removal, POP changes, serial radiographs (Figures 6-8), and ESR/CRP levels. There was significant pain reduction, and the sinus tract healed completely within a month. The K-wires were removed after eight weeks. Weight-bearing was encouraged starting at 10 weeks with the POP cast. The POP cast was removed after 12 weeks, and the patient was advised to wear footwear with medial arch support. The foot swelling resolved, and the patient is now symptom-free locally, with a healthy scar and a plantigrade foot (Figure 9) with mild hind foot valgus on weight bearing. The follow-up ESR and CRP levels were within normal limits. The patient completed a nine-month TB chemotherapy regimen and continued with six months of follow-up appointments.

## Discussion

In 2020, the majority of TB cases were concentrated in specific WHO regions: Southeast Asia (43%), Africa (25%), and the Western Pacific (18%). Smaller proportions were found in the Eastern Mediterranean (8.3%), the Americas (3.0%), and Europe (2.3%). Eight countries represented two-thirds of the global cases: India (26%), China (8.5%), Indonesia (8.4%), the Philippines (6.0%), Pakistan (5.8%), Nigeria (4.6%), Bangladesh (3.6%), and South Africa (3.3%) [2].

Tuberculous osteomyelitis is a rare infection, typically affecting the vertebrae, which accounts for around half of musculoskeletal TB cases. Involvement of the hand is less common, while skeletal TB of the foot is especially rare, with an occurrence rate of less than 0.13% among extrapulmonary cases [1]. During the initial *M. tuberculosis* infection, bacillemia can cause the spread of organisms to bone or synovial tissue. In most instances, small infection sites are controlled by local adaptive immune responses, keeping the infection subclinical.

Chronic joint swelling combined with constitutional symptoms should raise suspicion of skeletal TB. The condition is mainly managed with multidrug chemotherapy, but surgical procedures like biopsy, synovectomy, or debridement may be required in certain cases [3].

In the early stages, the primary purpose of surgery is to obtain a tissue diagnosis. While fine needle aspiration alone may not confirm the diagnosis, debridement with tissue biopsy can provide a conclusive diagnosis. Para-articular lesions with the potential to spread to nearby joints, especially in the midfoot, may require early debridement and biopsy to prevent further progression [3]. Additionally, certain chronic sinuses may need excision.

A limited number of articles and case reports on foot TB have been published, underscoring its rarity. Most of these publications emphasize biopsy and synovectomy, in addition to chemotherapy, as primary interventions [3]. Since all midfoot joints are interconnected, untreated osseous disease can easily spread across the foot, resulting in restricted movement with joint instability and poor functional outcomes. Prompt stabilization of joints in their proper alignment is crucial for preventing deformities [3]. Similar to the approach used in spinal TB, radical debridement and bone grafting can also be applied to the midfoot. Debridement helps reduce the infectious burden, while bone grafting offers a stable environment that minimizes the likelihood of disease recurrence [4]. Additionally, bone grafting facilitates joint fusion, resulting in pain relief and improved functional outcomes for the patient.

Dhillon et al. published four cases of cuboid osteomyelitis; only one required surgery due to a large osteolytic lesion with impending cuboid collapse. As the most important structure of the lateral column, it is crucial to prevent the cuboid from collapsing, particularly after curettage or debridement. In this case, a temporary external fixator was applied across the lateral column to maintain cuboid distraction until healing was observed. While curettage and primary bone grafting may not be ideal in the presence of active infection [3].

In our case, the scenario was different. The patient was initially started on ATT; however, symptoms persisted, accompanied by a discharging sinus despite the treatment. Imaging revealed subluxation of the calcaneocuboid joint with necrotic tissue present above the joint. To address this, we opted for a rational approach involving debridement of the necrotic tissue and bone, followed by joint fusion to ensure stability. This multimodal strategy resulted in a favorable outcome, with the resolution of constitutional symptoms and restoration of a good range of motion in the joint.

## Conclusions

Osteoarticular TB is primarily managed with antitubercular drugs, while surgical intervention aids in confirming the diagnosis and containing the disease’s spread to nearby joints. Similar to spinal TB treatment, debridement and bone grafting can be effective in managing foot TB, helping to prevent post-TB deformities. A high degree of clinical suspicion is essential in endemic regions, as TB affecting uncommon sites can easily lead to misdiagnosis and inappropriate treatment. Timely diagnosis and treatment are crucial to achieving better clinical outcomes in cases of skeletal TB.
